# A loss-of-function mutation in PTCH1 suggests a role for autocrine hedgehog signaling in colorectal tumorigenesis

**DOI:** 10.18632/oncotarget.1651

**Published:** 2013-12-11

**Authors:** Jon H. Chung, Fred Bunz

**Affiliations:** ^1^ Department of Radiation Oncology and Molecular Radiation Sciences, The Kimmel Cancer Center at Johns Hopkins, Baltimore, MD, USA

**Keywords:** Hedgehog, colorectal cancer, Patched, vismodegib, autocrine

## Abstract

Hedgehog (Hh) signaling is largely suppressed in the normal differentiated tissues of the adult but activated in many cancers. The Hh pathway can either be activated by the expression of Hh ligands, or by mutations that cause constitutive, ligand-independent signaling. Colorectal cancer cells frequently express Hh ligands that are believed to exert paracrine effects on the stromal component of the tumor. Evidence for a more direct role of Hh signaling on the growth and evolution of colorectal cancer cell clones has been lacking. Here, we report a loss-of-function mutation of PTCH1, a tumor suppressor in the Hh pathway, in a colorectal cancer that exhibits transcriptional upregulation of the downstream Hh gene GLI1. This finding demonstrates that autocrine Hh signaling can provide a selective advantage to evolving tumors that arise in the colorectal epithelia, and suggests a definable group of colorectal cancer patients that could derive enhanced benefit from Hh pathway inhibitors.

Recurrent mutations that cause Hh pathway activation are typically restricted to several cancer types, including basal cell carcinomas of the skin and medulloblastomas. Many other tumors that lack such driver mutations activate Hh signaling by expressing Hh ligands [1]. Tumor-expressed ligands can affect cancer cell growth in an autocrine fashion, but can also exert tumor-promoting paracrine effects on stromal cells.

Sonic hedgehog (Shh) and Indian hedgehog (Ihh) ligands are widely overexpressed in colorectal cancer-derived cell lines and in colorectal cancers, but the level of Hh pathway activity, as measured by the expression of the downstream effector GLI1, is highly variable [2, 3]. The lack of a positive correlation between ligand expression and Hh pathway activity suggests that autocrine growth stimulation may be absent in many ligand-expressing tumors. In contrast, Hh pathway activity in the stromal component of xenografted colorectal-derived tumors has been positively correlated with ligand expression by the tumor cells [3]. These studies support a model in which Hh ligands exert a predominantly paracrine effect in the majority of colorectal tumors, thereby contributing to tumor growth by processes such as angiogenesis [4]. It remains unclear if any colorectal tumor cells are directly responsive to Hh pathway activation.

Consistent with prior analyses of smaller colorectal cancer tumor panels [3], colorectal tumors comprehensively profiled in The Cancer Genome Atlas (TCGA; ref [5]) exhibit a wide range of GLI1 expression, suggesting that the level of Hh pathway activity may be highly variable in the cells that compose these tumors (Fig, panel A). Interestingly, the tumor with the highest level of GLI1 expression (tumor ID TCGA-AA-3715) harbored a missense mutation in *PTCH1* (*P681L*; c.2042C>T) that was identical to a confirmed somatic alteration previously identified in a basal cell carcinoma [6] (Fig, panel B). Because loss-of-function mutations in the tumor suppressor *PTCH1* cause elevated GLI1 expression in a large proportion of basal cell carcinomas [7, 8], we tested whether the P681L mutation altered PTCH1 function.

**Figure:**
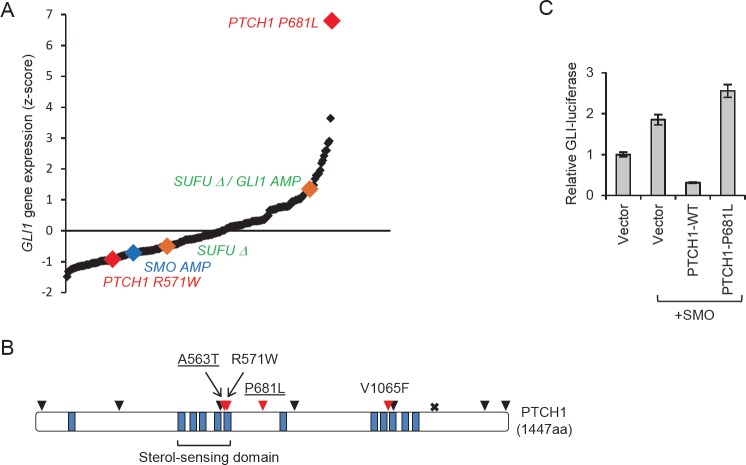
**A.** Expression of GLI1 in 296 colorectal tumors. Each point on the graph represents an individual tumor. Tumors were plotted in rank order according to the level of GLI1 expression. Red points indicate a tumor harboring a *PTCH1* mutation also found in at least one other tumor in the COSMIC database. The blue point indicates a tumor with *SMO* amplification. The green points indicate tumors with homozygous deletions involving SUFU, one of which also harbored a *GLI1* amplification.

**B.** Nonsynonymous *PTCH1* mutations in colorectal cancer (data from refs [5, 10]). The diagram shows the protein structure of PTCH1 with transmembrane domains marked in blue and the sterol-sensing domain indicated. The positions of non-synonymous single base substitutions are shown as triangles, a truncating mutation is indicated by an “x”. Mutated residues that were found in at least one other tumor in COSMIC are indicated in red. Mutations affecting A563 and P681 (underlined) were also found in basal cell carcinoma.

**C.** Loss-of-function in the *PTCH1 P681L* mutant. C3H10T1/2 cells were transfected with the indicated expression plasmids along with GLI-luciferase and Renilla luciferase reporters. The relative GLI-luciferase activity is shown for a triplicate experiment. Error bars indicate standard deviation.

In the canonical Hh signaling pathway, PTCH1 represses the activity of the G-protein coupled receptor SMO, while the GLI transcription factors are maintained in an inactive state by binding to SUFU [9]. The binding of Hh ligand to PTCH1 relieves the repression of SMO and thus reverses the inhibitory effect of SUFU on the GLI proteins. The activation of downstream GLI proteins causes increased expression of target genes, including *GLI1* and *PTCH1*. As expected, expression of exogenous *SMO* robustly activated a GLI-responsive luciferase (Gli-luc) reporter construct (Fig panel C). This activity was potently suppressed by expression of wild type *PTCH1*, but not *PTCH1 P681L*. Based on this functional defect and the marked elevation of *GLI1* expression in the tumor, we conclude that *PTCH1 P681L* is likely to be a driver mutation. The presence of *PTCH1* driver mutation in a colorectal cancer suggests that autocrine activation of Hh signaling can, in some cases, promote colorectal tumorigenesis.

*PTCH1* mutations that coded nonsynonymous amino acid changes were found in 4 percent (12/296) of the colorectal tumors that have been comprehensively profiled and curated [5, 10]. The tumors that harbored these mutations were globally hypermutated, and therefore among the approximately 15 percent of colorectal tumors that are mismatch repair deficient [11]. In the context of large numbers of passenger mutations, the impact of the majority of the *PTCH1* mutations was difficult to ascertain. Only *PTCH1 P681L* was strongly associated with elevated Hh pathway activation (increased GLI1 expression), and also recurrent in a type of cancer known to be initiated and maintained by autocrine Hh signals. A total of 1490 nonsynonymous mutations were detected in the tumor that harbored *PTCH1 P681L*, which also harbored well known driver mutations in *KRAS*, *BRAF* and *PIK3CA*. This tumor harbored no mutations or copy number alterations in *SMO* or *SUFU*, the other known driver genes in the Hh pathway.

Mutations that occur at a low frequency in a single type of cancer are more likely to be functionally relevant drivers if they recur in other cancers [12]. In addition to the *P681L* mutation, three additional nonsynonymous *PTCH1* mutations found in colorectal cancers occurred at codons that were also mutated in other cancers. *PTCH1 A563* was mutated in one colorectal cancer (p.A563T; c1687G>A), a lung adenocarcinoma (p.A563S; c1687G>T) and in a basal cell carcinoma (p.*A563*V; c1688C>T). R571 was mutated in one colorectal cancer (p.R571W; c1711C>T) and also in a T-cell acute lymphoblastic leukemia (p.R571Q; c1712G>A; ref [13]). V1065 was mutated in a colorectal cancer (p.V1065G; c.3194T>G; ref [10]) as well as in a lung carcinoma (*V1065F*; c.3193G>T). Gene expression data for the tumors harboring *PTCH1* mutations *A563T* and *V1065F* were not available. The tumor with a PTCH1 R571W mutation did not have elevated *GLI1* expression (Fig. panel A), suggesting that this mutation was a passenger rather than a driver.

In addition to *PTCH1*, *SMO* and *SUFU* were also mutated or otherwise altered in colorectal cancers at low frequency. Among the majority of the colorectal tumors that exhibited no mutational evidence of mismatch repair deficiency, two harbored homozygous deletions in *SUFU*, one of which occurred in a tumor that had also amplified *GLI1*. The latter tumor exhibited elevated GLI1 expression (approximately one standard deviation above the mean); the tumor harboring only the *SUFU* deletion did not exhibit elevated GLI1 expression (Fig. panel A). Amplification of *SMO* was found in one tumor, but this tumor did not exhibit elevated expression of SMO or GLI1 (Fig. panel A). Additional mutations found in *SUFU* and SMO were not recurrent, or were not associated with increased GLI1 expression (not shown).

Hh inhibitors have been successfully used to treat cancers that harbor mutations in the Hh pathway, but have been far less effective in tumors that are primarily driven by ligand overexpression [14]. Our observation in colorectal tumors of two *PTCH1* mutations, P681L and A563T/A563V, that have also been found in basal cell carcinomas suggests the existence of a small but perhaps not insignificant subset of colorectal tumors that grow in response to autocrine Hh signaling. Such tumors might be more likely to respond to therapeutic Hh inhibition. In a recent randomized phase II clinical trial involving 199 patients with metastatic colorectal cancer, addition of the Hh-inhibitor vismodegib (Erivedge, GDC-0449) to standard first-line therapy did not result in increased efficacy [15]. In that study, expression of Hh ligand, *SMO*, *GLI1* or *PTCH1* each failed to correlate with progression-free survival. In our analysis, the tumor that harbored the *PTCH1 P681L* mutation expressed GLI1 at a level that was greater than six standard deviations above the mean, and was therefore clearly an outlier (Fig A). Rare tumors with markedly elevated GLI1 expression might not have been represented in the cohorts thus far treated with vismodegib, or might have been inadvertently grouped with other tumors that were less dependent on autocrine signaling. We propose that routine genetic analysis of colorectal tumors would allow the identification of potentially responsive patients.

## METHODS

### Data mining and analysis

The Cancer Genome Atlas database containing the mutation, copy number and gene expression data and mutation data for colorectal tumors [5, 10] was accessed via cBioPortal [16] www.cbioportal.org/.

### Functional analysis of PTCH1

The wild type *PTCH1* plasmid pCI-PTCH1B-FLAG was obtained from Takashi Shimokawa. The *PTCH1*-*P681L* mutation was generated using the QuikChange II Site Directed Mutagenesis Kit (Agilent). C3H10T1/2 mouse cells were purchased from ATCC and grown in DMEM supplemented with 10 percent fetal bovine serum. Subconfluent cells were transfected with 200 ng of a 8XGli-luciferase reporter plasmid [17], 200 ng of the Renilla luciferase plasmid pGL4.74 (Promega), 200 ng SMO plasmid and 50 ng of the PTCH1 expression plasmids. Following transfection, each transfected well was split into three wells, and 24 h hours later shifted to DMEM with 0.5% fetal bovine serum for optimal activation of Hh signaling. Lysates were collected for analysis 72 h after transfection. Firefly luciferase and Renilla luciferase activity were measured using the Dual-Luciferase Reporter Assay System (Promega). Firefly luciferase values were divided by Renilla luciferase values to normalize for transfection efficiency.
